# Dr. Kentish's Letter

**Published:** 1808-04

**Authors:** E. Kentish

**Affiliations:** Bristol


### *To the Readers of the Medical and Physical Journal.*

Gentlemen,

An unfortunate accident, which nearly deprived me of existence, prevented me seeing the last Number of the Journal, so soon as I otherwise should have done; my medical friends during that period of danger, kindly kept it from my my view; nor will they even now allow me to occupy myself in answering it. I trust therefore, Gentlemen, that you will not permit your minds to be prejudiced by the malignant and calumnious aspersion thrown upon my character by Mr. Wood, (I presume it is Dr. J. Wood) of Newcastle; but that you will keep your judgments free, until I have an opportunity of giving you a statement of facts, which I trust will aquit me in your estimation, as firmly as I feel myself acquitted to my own honour.

I am, &c.

E. KENTISH, M. D.

*Bristol, March* 17, 1808.

